# Strategies of Intracellular Pathogens for Obtaining Iron from the Environment

**DOI:** 10.1155/2015/476534

**Published:** 2015-05-18

**Authors:** Nidia Leon-Sicairos, Ruth Reyes-Cortes, Alma M. Guadrón-Llanos, Jesús Madueña-Molina, Claudia Leon-Sicairos, Adrian Canizalez-Román

**Affiliations:** ^1^Unidad de Investigación de la Facultad de Medicina, Universidad Autónoma de Sinaloa, Cedros y Sauces, S/N Fracc. Fresnos, 80246 Culiacán, SIN, Mexico; ^2^Departamento de Investigación del Hospital Pediátrico de Sinaloa “Dr. Rigoberto Aguilar Pico”, Boulevard Constitución S/N, Colonia Jorge Almada, 80200 Culiacán, SIN, Mexico; ^3^Facultad de Medicina, Universidad Autónoma de Sinaloa, Cedros y Sauces, S/N Fracc. Fresnos, 80246 Culiacán, SIN, Mexico; ^4^Doctorado en Biotecnología, Facultad de Ciencias Químico-Biológicas, Universidad Autónoma de Sinaloa, Avenida de las Américas y Josefa Ortiz (Ciudad Universitaria), 80030 Culiacán, SIN, Mexico

## Abstract

Most microorganisms are destroyed by the host tissues through processes that usually involve phagocytosis and lysosomal disruption. However, some organisms, called intracellular pathogens, are capable of avoiding destruction by growing inside macrophages or other cells. During infection with intracellular pathogenic microorganisms, the element iron is required by both the host cell and the pathogen that inhabits the host cell. This minireview focuses on how intracellular pathogens use multiple strategies to obtain nutritional iron from the intracellular environment in order to use this element for replication. Additionally, the implications of these mechanisms for iron acquisition in the pathogen-host relationship are discussed.

## 1. Introduction

Intracellular pathogens are organisms that are capable of growing and reproducing inside host cells. These pathogens can be divided into facultative intracellular parasites and obligate intracellular parasites [1]. Intracellular microorganisms are very important because they cause many human diseases, resulting in significant morbidity and mortality. Some examples of infectious diseases of global importance that are caused by intracellular microorganisms include tuberculosis, leprosy, typhoid, listeriosis, Legionnaire's disease, malaria, leishmaniasis, Chagas' disease, and toxoplasmosis. The course of infection is frequently long lasting and eventually results in chronic disease [2–4]. Facultative intracellular parasites, for example, bacteria such as* Francisella tularensis*,* Listeria monocytogenes*,* Salmonella typhi*,* Mycobacterium *spp., and* Neisseria meningitidis*, are capable of living and reproducing either inside or outside host cells. Obligate intracellular parasites cannot reproduce outside their host cell, which means that the parasite's reproduction is entirely reliant on intracellular resources. Obligate intracellular parasites that infect humans include all viruses; certain bacteria such as* Chlamydia* and* Rickettsia*; certain protozoa such as* Trypanosoma* spp.,* Plasmodium*, and* Toxoplasma*; and fungi such as* Pneumocystis jirovecii* [3]. Facultative intracellular bacteria invade host cells when they can gain a selective advantage in the host. Bacteria that can enter and survive within eukaryotic cells are shielded from humoral antibodies and can be eliminated only by a cellular immune response [5]. Moreover, once inside host cells, bacteria must utilize specialized mechanisms to protect themselves from the harsh environment of the lysosomal enzymes encountered within the cells. Some examples include the bacterium* Legionella pneumophila*, which prefers the intracellular environment of macrophages for growth so it induces its own uptake and blocks lysosomal fusion by an undefined mechanism [6];* Rickettsia*, which destroys the phagosomal membranes (with which the lysosomes fuse); and* Salmonella* and* Mycobacterium* spp., which are resistant to intracellular killing by phagocytic and other cells [2]. Other facultative intracellular bacteria include enteroinvasive* Escherichia coli*,* Listeria monocytogenes, Neisseria* spp., and* Shigella* spp. [2, 7].

Obligate intracellular bacteria cannot live outside the host cell. Chlamydial cells are unable to carry out energy metabolism and lack many biosynthetic pathways and therefore are entirely dependent on the host cell to supply them with ATP (adenosine triphosphate) and other intermediate molecules [8]. Obligate intracellular bacteria cannot be grown in artificial media (agar plates/broths) in laboratories but require viable eukaryotic host cells (e.g., cell culture, embryonated eggs, and susceptible animals). Additional obligate intracellular bacteria include* Coxiella burnetii*,* Rickettsia* spp., and others [8, 9].

Microbial access to host nutrients is a fundamental aspect of infectious diseases. Pathogens face complex dynamic nutritional host microenvironments that change with increasing inflammation and local hypoxia. Because the host can actively limit microbial access to its nutrient supply, pathogens have evolved various metabolic adaptations to successfully exploit available host nutrients to facilitate their own proliferation [10]. Iron (Fe) is a key global regulator of cellular metabolism, which makes Fe acquisition a focal point of the biology of pathogen systems. In the host environment, the success or failure of Fe uptake processes impacts the outcome of pathogenesis [11]. After phagocytosis by macrophages, intracellular bacteria are located in a membrane-bound vacuole (phagosome), but the ensuing trafficking of this vacuole and subsequent bacterial survival strategies vary considerably. If the ingested bacteria have no intracellular survival mechanisms, the bacteria-containing phagosomes fuse with the lysosomal compartment, and bacteria are digested within 15–30 min. For this reason, the majority of intracellular bacteria and other parasites must keep host cells alive as long as possible while they are reproducing and growing [7, 9]. To grow, intracellular pathogens need nutrients such as the iron, that might be scarce in the cell, because this is usually retained or stored by proteins.

Pathogens that infect macrophages require Fe for growth, but, during infection, Fe is required by both the host cell and the pathogen that inhabits the host cell [12]. Macrophages require Fe as a cofactor for the execution of important antimicrobial effector mechanisms, including the NADPH- (nicotinamide adenine dinucleotide phosphate-oxidase-) dependent oxidative burst and the production of nitrogen radicals catalyzed by the inducible nitric oxide synthase [13]. On the other hand, intracellular bacteria such as* Legionella pneumophila*,* Coxiella burnetii*,* Salmonella typhimurium*, and* Mycobacterium tuberculosis* have an obligate requirement for Fe to support their growth and survival inside host cells [14]. In fact, it has been documented that deprivation of Fe* in vivo* and* in vitro* severely reduces the pathogenicity of* M. tuberculosis*,* C. burnetii*,* L. pneumophila*, and* S. typhimurium* [13–15].

## 2. Iron in the Human Host

Iron (Fe) is essential for the growth of all organisms. The human body contains 3–5 g of Fe distributed throughout the body in the protein hemoglobin, tissues, muscles, bone marrow, blood proteins, enzymes, ferritin, hemosiderin, and transport in plasma. Iron (approximately 75%) is contained in the protein hemoglobin (Hb) and in other iron-bound proteins that are important for cellular processes, and whatever remains in plasma (approximately 25%) is bound to plasma proteins such as transferrin (Tf) [16].

Dietary Fe has two main forms: heme and nonheme. Plants and iron-fortified foods contain nonheme Fe only, whereas meat, seafood, and poultry contain both heme and nonheme iron. Heme iron, which is formed when Fe combines with protoporphyrin IX, contributes about 10% to 15% of total Fe intakes in western populations [17]. Intestinal absorption is the primary mechanism regulating Fe concentrations in the body. Once ingested, Fe absorption occurs predominantly in the duodenum and upper jejunum. The mechanism of iron transport from the gut into the blood stream remains unknown. The first step of the pathway of iron absorption in the human host involves reduction of ferric Fe^3+^ to Fe^2+^ in the intestinal lumen by reductases or cytochrome b and transport of Fe^2+^ across the duodenal epithelium by the apical transporter DMT1 (divalent metal transporter). In nonintestinal cells most Fe uptake occurs via either the classical clathrin-coated pathway utilizing transferrin receptors or the poorly defined transferrin receptor independent pathway. Tf is the principal Fe storage protein that stores and releases Fe inside cells that express the transferrin receptor (TfR). The delivery of Fe from Tf is mediated by an acidic pH 5.5 of the endocytic vesicles carrying holo-Tf and TfR complexes. Fe is then transported across the endosomal membrane and utilized. Excess intracellular Fe is sequestered into the protein Ft [18, 19].

In a healthy individual Fe is largely intracellular, sequestered within Ft or as a cofactor of heme complexed to Hb within erythrocytes. Any extracellular free Fe is rapidly bound by circulating Tf. Hb or heme that is released as a result of natural erythrocyte lysis is captured by haptoglobin and hemopexin, respectively. Taken together, these factors ensure that vertebrate tissue is virtually devoid of free iron [21]. Maintaining cellular Fe content requires precise mechanisms for regulating its uptake, storage, and export. The iron response elements or iron-responsive elements (IRP1 and IRP2) are the principal regulators of cellular Fe homeostasis in vertebrates. IRPs are cytosolic proteins that bind to Fe-responsive elements (IREs) in the 5′ or 3′ untranslated regions of mRNAs encoding proteins involved in Fe uptake (TfR1, DMT1), sequestration (H-ferritin subunit (FTH1) and L-ferritin subunit (FTL)), and export (ferroportin). When cells are Fe deficient, IRPs bind to 5′ IREs in ferritin and ferroportin mRNAs with high affinity to repress translation and to 3′ IREs in TfR1 mRNA to block its degradation (Tf is involved in the transport or Fe). When Fe is in excess, IRPs do not bind to IREs, increasing synthesis of Ft and ferroportin (proteins involved in the storage of Fe), while promoting the degradation of TfR1 mRNA. The coordinated regulation of Fe uptake, storage, and export by the IRPs ensures that cells acquire adequate Fe for their needs without reaching toxic levels [22].

**Figure 1:**
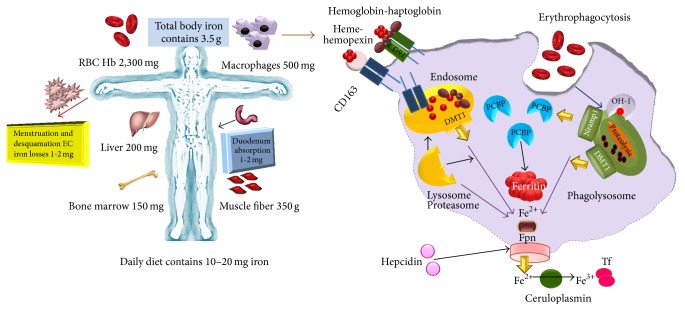
Iron content in the human body and iron-containing proteins in a macrophage. The average male adult contains approximately 3.5 g of iron. Approximately 2 g of iron is in hemoglobin: 1 g in body is stored predominantly in the liver and the rest in myoglobin and other iron-containing proteins. Approximately 1 to 2 mg of iron is lost each day by epithelial shedding in the gastrointestinal tract and the skin and through blood loss in menstruating women. Western diets contain a much greater amount of iron (10 to 20 mg) than what is absorbed daily under normal circumstances (1 to 2 mg). The macrophage is a key agent in iron homeostasis as well as in inflammatory hypoferremia. Macrophages in the spleen and in the liver (Kupffer cells) and perhaps elsewhere recognize damaged or senescent erythrocytes, phagocytize them, and digest them to extract heme and eventually iron. Macrophages can also scavenge heme and hemoglobin, usually complexed with hemopexin and haptoglobin, respectively, and endocytosed by CD163 and CD91, respectively. Whether phagocytosed in erythrocytes or endocytosed by scavenging, hemoglobin undergoes proteolysis to release heme. Heme is degraded by HO-1 to release iron, which is exported to the cytoplasm by DMT1 and probably also by Nramp1. Cytoplasmic chaperones family deliver iron for storage in the protein ferritin. Alternatively, iron from endosomes or phagolysosomes may also be delivered by an unknown carrier to ferroportin (Fpn) for export [20].

The ability of pathogens to obtain Fe from Tf, Lf, Ft, Hb, and other iron-containing proteins of their host is central to whether they live or die [14]. This is because these proteins are the main Fe sources for intracellular pathogens in the macrophage. Iron homeostasis in the macrophage is determined by uptake processes through Lf, Tf, DMT-1, and phagocytosis of senescent erythrocytes as well as by export through ferroportin (Fpn), as we have discussed before. Inside infected macrophages, a pathogen's access to Fe may be limited by natural resistance-associated macrophage protein 1 (SLC11A1, formerly Nramp1). SLC11A1 is a divalent metal transporter, recruited to the late endosomal and phagosomal membrane of macrophages and other professional phagocytes. Although SLC11A1 contributes to macrophages' efficiency in the recycling of erythrocyte-derived Fe, the main function of SLC11A1 seems to be the protection against microbes [20]. Its gene is present in inbred strains of mice in two allelic forms that determine the resistance or susceptibility to several intracellular pathogens such as* Mycobacterium* spp.,* Salmonella* spp., and* Leishmania* spp. [23]. Some groups of researchers have suggested that Fe is transported via this protein into the pathogen-containing phagosome, causing the death of the pathogen by catalyzing the formation of reactive oxygen species (ROS), while others argue for Fe efflux from the phagosome, restricting pathogenic growth by Fe deprivation [23, 24]. Another Fe transporter that is expressed in macrophages is Fpn. This transporter is present in the macrophage cytoplasmic membrane and is responsible for Fe export. Overexpression of Fpn has been reported to inhibit the intramacrophagic growth of* M. tuberculosis* and* Salmonella enterica*, presumably through Fe deprivation. The details of this mechanism are unclear [25, 26]. A scheme of Fe sources in the human body and iron homeostasis inside the macrophage is shown in Figure 1.

## 3. Mechanisms Used by Intracellular Pathogens for Obtaining Iron: A General Point of View

During infection, pathogens are capable of altering the battlefield to increase the abundance of potential Fe sources. For example, bacterial cytotoxins damage host cells, leading to the release of Ft, while hemolytic toxins from bacteria can lyse erythrocytes, liberating Hb. The resulting inflammatory response includes the release of Lf from secondary granules contained with polymorphonuclear leukocytes (PMNs) [10, 21, 27]. Pathogens are capable of exploiting these diverse Fe sources through the elaboration of a variety of Fe acquisition systems. In the case of extracellular pathogens, they can acquire Fe through receptor-mediated recognition of Tf, Lf, hemopexin, hemoglobin, or hemoglobin-haptoglobin complexes [19, 27]. Alternatively, secreted siderophores can remove Fe from Tf, Lf, or Ft, whereupon siderophore-iron complexes are recognized by cognate receptors at the bacterial surface. Siderophores are small ferric iron chelators that bind with extremely high affinity (iron formation constants *K*_*d*_ range from 10−20 to 10−50 M), some of which can extract iron from Tf and Lf [21]. Analogously, secreted hemophores can remove heme from Hb or hemopexin and deliver heme to bacterial cells through binding with hemophore receptors. Siderophore mediated Fe acquisition is inhibited by the innate immune protein siderocalin, which binds siderophores and prevents receptor recognition. This host defense is circumvented through the production of stealth siderophores that are modified in such a way as to prevent siderocalin binding [21, 27].

**Figure 2:**
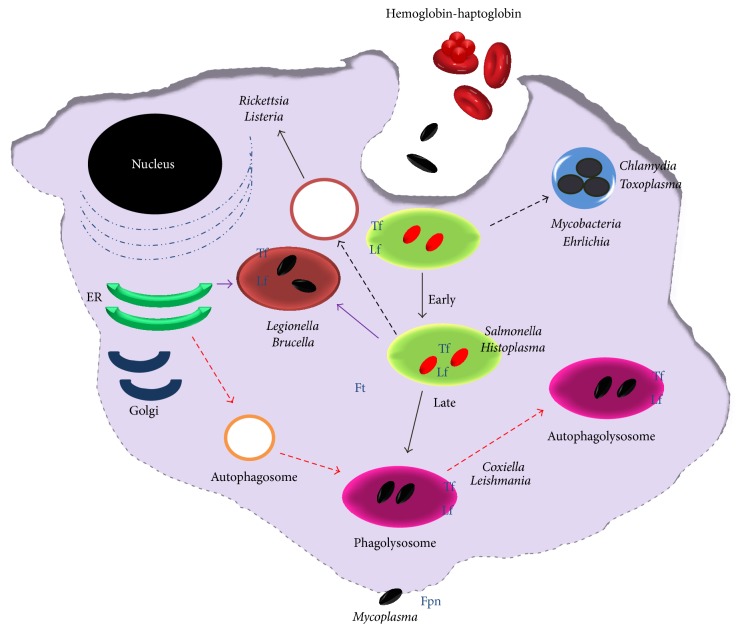
Intracellular parasites and iron sources inside of the macrophage. During infection with intracellular bacteria, iron is required by both the host cell and the pathogen that inhabits the host cell. Macrophages require iron as a cofactor for the execution of important antimicrobial effector mechanisms, and so forth. On the other hand, intracellular bacteria also have an obligate requirement for iron to support their growth and survival inside cells. Some pathogens are internalized into membranous compartments (endosomes/phagosome) and then subsequently trafficked to the lysosome for degradation. Intracellular pathogens have evolved specific mechanisms to survive within this intracellular environment, for example,* Salmonella* persists in the endocytic pathway, and others escape the endo-/lysosomal system and exist in the cytosol. Other bacteria remain within a membranous envelope that may be a modified version of the endoplasmic reticulum (*L. pneumophila*) or a membranous compartment generated by the bacteria (*Chlamydia*), and so forth. Intracellular pathogens can acquire iron because macrophages contain iron-proteins such as transferrin, lactoferrin, ferritin, hemopexin, hemoglobin, or hemoglobin-haptoglobin complexes, in its different compartments.

For proper use of Fe, extracellular or intracellular parasites must possess at least the following systems: (a) Fe sensors for monitoring Fe concentration in the intracellular environment, (b) synthesis and release of high-affinity compounds that can compete with host Fe binding proteins for Fe acquisition and storage, or proteases to degrade these host Fe binding proteins, (c) transportation of these Fe-loaded molecules and their assimilation, and (d) regulation of the expression of proteins involved in iron metabolism, in order to maintain iron homoeostasis [27, 28]. Once ingested by macrophages, many intracellular parasites are taken up by phagosomes through endocytosis. Thus, the success of intracellular parasites seems to be related mainly to their ability to take up Fe from the proteins Tf, Hb, hemoglobin-haptoglobin, free heme, and Ft.  Figure 2 shows intracellular parasites and Fe sources inside a macrophage.

In order to take the Fe from Tf, these systems can be divided into three main categories: siderophore-based systems, heme acquisition systems, and transferrin/lactoferrin receptors.

Upon removing Fe from host proteins, iron-loaded siderophores are bound by cognate receptors expressed at the bacterial surface. The siderophore-iron complex is then internalized into the bacterium and the Fe is released for use as a nutrient source [21]. Heme acquisition systems typically involve surface receptors that recognize either heme or heme bound to hemoproteins such as hemoglobin or hemopexin. Heme is then removed from hemoproteins and transported through the envelope of bacteria into the cytoplasm. Once inside the cytoplasm, the iron is released from heme through the action of heme oxygenases or reverse ferrochelatase activity. Bacterial pathogens can also elaborate secreted heme-scavenging molecules that remove heme from host hemoproteins. These molecules, known as hemophores, are functionally analogous to siderophores but are proteins that target heme, whereas siderophores are small molecules that target iron atoms [29]. In addition to acquiring Fe from Tf and Lf through siderophore-based mechanisms, some pathogens are capable of direct recognition of these host proteins through receptors [21]. These receptors are modeled to recognize Tf or Lf, leading to Fe removal and subsequent transport into the bacterial cytoplasm. Additionally, acidification of the phagosome permits Fe release from Tf and probably Lf and, in this way, some pathogens can gain access to this element directly [19, 21, 30].

The following sections summarize the Fe acquisition systems used by some intracellular pathogens.  Table 1 shows Fe sources, mechanism of uptake, transport and regulation, used by intracellular parasites.

## 4. Mechanism of Intracellular Pathogens for Obtaining Iron from Host Sources

### 4.1. *Francisella tularensis*

*F. tularensis*, the bacterial cause of tularemia, is a virulent intracellular pathogen that can replicate in multiple cell types. Acidification of the phagosome and acquisition of Fe is essential for growth of* F. tularensis* [31]. An acidic pH promotes the release of Fe from host cell Tf. To acquire the Fe from Tf,* F. tularensis* involves a receptor for this protein (Transferrin receptor 1, TfR1), induction of ferrireductases, an iron membrane transporter (DMT-1), and iron regulatory proteins (IRP1 and IRP2); this is an active Fe acquisition system associated with a sustained increase of the labile Fe pool inside the macrophage [31]. In addition,* F. tularensis* uses high-affinity transportation of ferrous Fe across the outer membrane via the proteins FupA and FslE. FsIe appears to be involved in siderophore-mediated ferric Fe uptake, whereas FupA facilitates high-affinity ferrous Fe uptake [32]. It has been hypothesized that* F. tularensis* uses the Fe from Lf to sustain its growth; however, the mechanism of Fe acquisition from LF remains undetermined [33]. It is most likely that* F. tularensis* can infect many types of cells because it contains several strategies for Fe acquisition. It has been reported that the expression of certain* F. tularensis* virulence genes is clearly regulated by Fe availability [34].

The expression of TfR1 is critical for the intracellular proliferation of* Francisella*. This contrasts with infection of macrophages by* Salmonella typhimurium*, which does not require expression of TfR1 for successful intracellular survival. Macrophages infected with* Salmonella* lack significant induction of DMT-1, Steap3, and IRP1 and maintain their labile Fe pool at normal levels [12]. Authors argue that this might be explained by* Salmonella's* intracellular localization within an endosomal structure or perhaps by more efficient Fe acquisition strategies compared to* Francisella* [12].

### 4.2. *Salmonella* spp

*Salmonella typhimurium* is an invasive pathogen that causes diseases ranging from mild gastroenteritis to enteric fever. To establish a systemic infection,* Salmonella* spp. must invade the epithelial wall of the intestine before the bacteria are ingested by immune effector cells and transported to lymph nodes, the spleen, and other organs.* Salmonella *spp. reside within modified phagosomes in macrophages, where replication is promoted and killing is evaded. Fe is an essential micronutrient for replication, and* Salmonella* spp. harbor various Fe acquisition systems, such as the siderophores enterobactin and salmochelin [35]. As iron sources,* Salmonella* spp. use Fe^2+^, Fe^3+^, heme, ovotransferrin, and Tf [35, 36].* S. Typhimurium* acquires Fe^2+^ from hemophagocytic macrophages and also secretes siderophores via IroC and EntS to bind Fe^3+^, which is subsequently taken up by outer membrane receptors including IronN and FepA. ABC transporters such as FepBCDG are responsible for the transport of siderophores through the cytoplasmic membrane, whereas molecular iron is taken up via Feo-mediated transmembrane transport [35, 36].

During the infection process* in vivo*,* S. typhimurium* induces a number of virulence genes that are required to circumvent host defenses and/or acquire nutrients from the host. A putative Fe transporter in* Salmonella* called Pathogenicity Island 1, or sitABCD, has been characterized. The sitABCD operon is induced under Fe-deficient conditions* in vitro* and is repressed by Fur (ferric uptake regulator). This locus is specifically induced in animal models after invasion of the intestinal epithelium, suggesting that SitABCD plays an important role in Fe acquisition in the animal. To regulate its Fe content,* Salmonella enterica* serovar Typhimurium possesses four ferritins: bacterioferritin (Bfr), ferritin A (FtnA), ferritin B (FtnB), and Dps. The heme-containing Bfr accounts for the majority of stored Fe, followed by FtnA. Inactivation of Bfr elevates the free intracellular Fe concentration and enhances susceptibility to H_2_O_2_ stress. The DNA-binding Dps protein provides protection from oxidative damage without affecting the free intracellular Fe concentration at steady state. FtnB appears to be particularly important for the repair of Fe-sulfur clusters of aconitase that undergo oxidative damage, and, in contrast to Bfr and FtnA, is required for* Salmonella* virulence in mice. Moreover, FtnB and Dps are repressed by the Fe-responsive regulator Fur and induced under conditions of Fe limitation, whereas Bfr and FtnA are maximally expressed when Fe is abundant. The absence of a conserved ferroxidase domain and the potentiation of oxidative stress by FtnB in some strains that lack Dps suggest that FtnB serves as a facile cellular reservoir of Fe^2+^ [37].

### 4.3. *Chlamydia* spp

Chlamydia is an infection that is caused by the bacteria* Chlamydia trachomatis*. It is the most common sexually transmitted disease in the U.S., with nearly 3 million cases reported each year (the actual number of cases is likely much higher). The developmental cycle of* C. trachomatis* includes two forms: an infectious elementary body (EB) and a reticulate body that multiplies within the inclusion by binary fission. A third developmental form is the persistent form, which exists as a mechanism of survival under stressful conditions. Persistence is induced in response to changes in the culture medium, including amino acid or Fe deprivation, and in the presence of antibiotics or cytokines such as gamma interferon (IFN) [38]. It has been shown that Fe is an essential factor in the growth and survival of* C. trachomatis* and* C. pneumoniae* (this bacterium causes pneumonia) [39]. Although homologues for bacterial siderophores are missing in the genome of this bacterium, TfR expression does occur.* C. trachomatis* also appears to be missing a tonB analogue, which would span the periplasm and is crucial in energy transfer to substrate-specific outer membrane transporters that are used to bring Fe-siderophore complexes to the cell. Considering these apparent gaps in the genome, one could speculate that the* C. trachomatis* genome would need a reductase on the inclusion membrane to transport Fe^2+^ from the eukaryotic cytosol into the inclusion.* C. trachomatis* and* C. pneumoniae* appear to use the host's Fe transport pathways by attracting TfR and Ft to the phagosome [39]. A report from Vardhan et al. (2009) showed that* C. trachomatis* alters the Fe-regulatory protein-1 (IRP-1) binding capacity and modulates cellular iron homeostasis in HeLa-229 cells, suggesting that Fe homeostasis is modulated in CT-infected HeLa cells at the interface of acquisition and commensal use of Fe [40].

ATP-binding cassette (ABC) transport systems play a role in the acquisition of Fe and Fe-complexes, amino acids, sugars, and other compounds. They consist of a soluble periplasmic protein that binds the targeted molecule and changes conformation to close around the substrate. The periplasmic binding protein moves to and binds the transmembrane protein permease in receptor-ligand mechanisms. An ATP-binding lipoprotein binds to the ATP, creating a conformational change in the permease complex that transports the substrate into the cytoplasm. In other pathogenic bacteria, ABC transport systems that transport Fe, zinc, and manganese into the cytoplasm include Tro from* Treponema pallidum*, Yfe from* Yersinia pestis*, and Fbp from* Neisseria meningitidis* [40]. There is evidence that YtgA secretion occurs in* C. trachomatis*, and YtgA does have high homology with periplasmic binding proteins of the ABC transport systems.* yta*A is a gene of 978 bp that resides in an operon with* ytg*BVD. YtgB and Ytg have predictable membrane-spanning domains and most likely form the pore of the ABC transporter. YtgA contains similar metal-binding motifs (e.g., histidine, tyrosine) to other metal-binding periplasmic proteins, suggesting a role for YtgA as an Fe-binding periplasmic protein, in addition to its location on the chlamydial membrane [41].

### 4.4. *Neisseria* spp

Acquisition of Fe and Fe-complexes has long been recognized as a major determinant in the pathogenesis of* Neisseria* spp., and some of their high-affinity iron uptake systems are important virulence factors in bacteria. These have been shown to play a major role in promoting the survival of the meningococcus within the host. Most species are Gram-negative bacteria that are primarily commensal inhabitants or reside in the mucus membranes of mammals. There are 12* Neisseria* species of human origin, with* N. meningitidis* and* N. gonorrhoeae* being important opportunistic pathogens. These intracellular pathogens contain high-affinity iron uptake systems, which allow meningococci to utilize the human host proteins Tf, Lf, Hb, and haptoglobin-hemoglobin as sources of essential Fe [29, 42]. Although the meningococci do not produce siderophores, studies indicate that meningococci may be able to use heterologous siderophores secreted by other bacteria. For some time, it has been reported that the gonococci could utilize ferric enterobactin, enterobactin derivatives, aerobactin, and salmochelin S2 in a FetA- and TonB-dependent manner [29]. In* N. gonorrhoeae*, an outer membrane protein named FetA (formerly FrpB) was recently described. FetA is an outer membrane transporter and is part of an iron-regulated operon that encodes a periplasmic binding protein and the components of a putative ABC transport system. FetA has demonstrated low binding affinity and the transport of ferric enterobactin. The binding contact of FetA for enterobactin was much lower than that for other enterobactin receptors, and it was therefore proposed that this receptor could interact with high affinity to an as-yet unidentified phenolate siderophore. A homologous protein, with 91% similarity to gonococcal FetA, has been identified in* N. meningitidis* and presumably functions in a similar manner [30, 43]. Only* fet*A and not the downstream genes require an iron-regulator MpeR for regulation. MpeR regulation is important because it may aid in gonococcal immune evasion. MpeR was suggested to modulate any change in* mtr*F expression that is needed for full hydrophobic agent resistance. AraC-like regulators of* N. meningitidis* are homologues of the* N. gonorrhoeae* type MpeR that is specific to the pathogenic* Neisseria* species. Both are induced during Fe limitation, and this regulation is also mediated by the Fur regulator. The presence of MpeR in a regulatory cascade downstream of the Fur master Fe regulator suggests that it is being expressed in the Fe limiting environment of the host, where it may in turn regulate a group of genes, including the divergent Fe transport locus, in response to signals that are important for infection [44].

Two proteins, transferrin-binding protein A (TbpA) and transferrin-binding protein B (TbpB), function as the transferrin receptor in* N. meningitidis*. TbpA and TbpB are induced along with several other proteins in the outer membranes of* N. meningitidis* under Fe-restricted conditions [30]. Initially, an affinity isolation procedure using biotinylated transferrin was employed to demonstrate the presence of two transferrin-binding proteins in* N. meningitidis*. The proteins that bound transferrin were TbpA (formerly Tbp1), which is 98 kDa, and TbpB (formerly Tbp2), which is 68 kDa [45]. Among different meningococcal isolates, the molecular masses of TbpA and TbpB vary, with TbpA ranging from 93 to 98 kDa and the more heterogenetic TbpB varying from 68 to 85 kDa. TbpA can be found in all strains. Although it has not been characterized as well as the Tf receptor, the Lf receptor is believed to be an important meningococcal virulence factor [29]. The Lf receptor of* N. meningitidis*, like the Tf receptor, consists of two protein components, LbpA and LbpB. Initial experiments using affinity isolation by Lf identified a 98-kDa lactoferrin-binding protein named LbpA, formerly known as IroA [46].

### 4.5. *Legionella pneumophila*

*Legionella pneumophila*, the causative agent of Legionnaire's disease, is a facultative intracellular parasite of human macrophages and freshwater amoebae. This pathogenic bacterium is commonly found in water, thereby presenting a risk that it could be transmitted to humans via inhalation of contaminated aerosols.* L. pneumophila* resides in the phagosome, although this phagosome does not fuse with endosomes and lysosomes and is at nearly neutral pH during the early stages of the intracellular life cycle. It appears to fuse with low-pH cellular compartments during the later stages of the infection [47].

The ability of* L. pneumophila* to acquire host cell Fe is pivotal for the parasite to establish a successful intracellular infection. To occupy its intracellular niche, this pathogen has developed multiple Fe acquisition mechanisms: the ira AB locus, which encodes a transporter for Fe-loaded peptides; the cytochrome c maturation ccm genes; the Fe-regulated frgA, whose product is homologous to aerobactin synthetases; legiobactin siderophores; and two internal ferric reductases. Robey and Cianciotto (2002) identified and characterized* L. pneumophila* Feo AB, which bears homology to* E. coli* and* Salmonella enterica* serovar Typhimurium FeoAB. In those bacteria, FeoB has been shown to be a ferrous Fe transporter and FeoA is possibly involved in Fe^2+^ uptake [48].

In 2014, Portier and Cols discovered gene ipp_2867, which was highly induced in Fe-restricted conditions. A sequence analysis predicts that Lpp_2867 is a membrane protein involved directly or indirectly in Fe^2+^ transport and is also a virulence factor [49].

### 4.6. *Shigella* spp

*Shigella* is a Gram-negative bacterium of the Enterobacteriaceae family and is the etiological agent of bacillary dysentery or shigellosis.* Shigella* encompasses four subgroups (*S. flexneri*,* S. sonnei*,* S. dysenteriae*, and* S. boydii*), and all species are able to grow in a variety of environments, including intracellularly in host epithelial cells.* Shigella* has a number of different Fe transport systems that contribute to the bacterium's ability to grow in these diverse environments [50]. Siderophore Fe uptake systems, heme transporters, and Fe^3+^ and Fe^2+^ transport systems are present in these bacteria, and the genes encoding some of these systems appear to have spread among the* Shigella* species by horizontal transmission [50, 51]. Fe is not only essential for the growth of* Shigella* but also plays an important role in the regulation of metabolic processes and virulence determinants in* Shigella*. This regulation is mediated by the repressor protein Fur and the small RNA RyhB [52]. The only Fe transport system that appears to be common to all members of the* E. coli*/*Shigella* group is Feo.* Shigella* spp. have transport systems for both ferric and ferrous iron. The Fe can be taken up as free Fe or complexed with a variety of carriers. All* Shigella* species have both the Feo and Sit systems for acquisition of Fe^2+^, and all have at least one siderophore-mediated system for transport of Fe^3+^ [53]. Several of the transport systems, including Sit, Iuc/IutA (aerobactin synthesis and transport), Fec (ferric di-citrate uptake), and Shu (heme transport), are encoded within pathogenicity islands. The presence and the genomic locations of these islands vary considerably among the* Shigella* species and even between isolates of the same species [53, 54]. The expression of the Fe transport systems is influenced by the concentration of Fe and by environmental conditions, including the level of oxygen. ArcA and FNR regulate Fe transport gene expression as a function of oxygen tension, with the sit and iuc promoters being highly expressed in aerobic conditions, while the feo Fe^2+^ transporter promoter is most active under anaerobic conditions [52]. The effects of oxygen are also observed in infection of cultured cells by* S. flexneri*; the Sit and Iuc systems support plaque formation under aerobic conditions, whereas Feo allows plaque formation to occur anaerobically [52, 53].

### 4.7. *Listeria monocytogenes*

*L. monocytogenes* is a Gram-positive, intracellular pathogen responsible for the fatal disease listeriosis.* L. monocytogenes* is recognized as a significant public health problem. The ability of this bacterium to acquire and utilize Fe is not only essential during infection but can also support its growth and survival in many diverse environmental niches.

*L. monocytogenes* possesses at least 4 mechanisms that enable Fe uptake: (1) acquisition of protein-bound Fe that involves the HupDGC protein (for the uptake of hemin, hemoglobin), or Fhu protein (involved in the uptake of ferrichrome siderophores); inside the cell, then Fe can be bound to the Fri protein (ferritin-like) Fur regulated; (2) extracellular and/or surface-bound Fe reductases; (3) a citrate inducible ferric citrate uptake system; and (4) siderophore and siderophore-like systems [55].

The* Listeria* life cycle involves escape from the phagosome, which is considered to be Fe-limiting and permits proliferation in the host-cell cytosol, where Fe-saturated Ft is stored. It has been hypothesized that* L. monocytogenes* has access to Fe through increased expression of the PrfA-regulated virulence factors listeriolysin (LLO) and ActA, which are used for phagosomal escape. Increased Fe concentrations result in the upregulation of internalin proteins InlA and InlB, which are required for invasion [56].

Fe homeostasis in* Listeria* is controlled by the regulatory protein Fur. It has been shown that expression of Fur is negatively regulated by PerR, a Fur homologue that is involved in the oxidative stress response. Fourteen Fur-regulated genes have been identified in* L. monocytogenes*, including genes that encode Fe^2+^ transporters and ferrichrome ABC transporters and proteins involved in Fe storage [56, 57].

### 4.8. *Coxiella burnetii*

*Coxiella burnetii* is the causative bacterial agent of Q fever in humans and is one of the most infectious pathogens known. Human infection with* C. burnetii* is generally a zoonosis that is acquired by inhalation of contaminated aerosols. Q fever typically presents as an acute, self-limiting flu-like illness accompanied by pneumonia or hepatitis. In 1% of cases, a severe chronic infection can occur, in which endocarditis is the predominant manifestation [58]. It is essential for most pathogenic bacteria to overcome the limitation of Fe in the intracellular host. To overcome this limitation, bacteria maintain cell storage systems under the tight control of Fur. It has been suggested that it is an absolute requirement for* C. burnetii*, similar to* L. pneumophila*, to regulate Fe assimilation via the Fur regulon. One study revealed that the Fur-regulon in* C. burnetii* consists of a Fur-like protein (CBU1766) and the putative iron-binding protein Frg1 (CBU0970) [59].

Iron plays a rather limited role in the pathogenesis of* C. burnetii*. Reports have described the expression of a thiol-specific peroxidase (CBU0963) in* C. burnetii* that belongs to the atypical 2-cysteine subfamily of peroxiredoxins, also designated as bacterioferritin comigratory proteins (BCPs). The implication is that this protein might protect DNA from the Fenton reaction [60]. Comparison to* L. pneumophila*, a phylogenetic relative, revealed that* C. burnetii* rarely encodes any known Fe acquisition or storage proteins, aside from some Fe dependent pathways, as well as the heme biosynthesis pathway and proteins such as SodB.

### 4.9. *Mycobacterium* spp

Mycobacterium is a genus of Actinobacteria, given its own family, the Mycobacteriaceae. The genus includes pathogens known to cause serious diseases in mammals, including tuberculosis (*Mycobacterium tuberculosis*) and leprosy (*Mycobacterium leprae*). Similar to most microorganisms,* Mycobacterium tuberculosis*, the causative agent of tuberculosis, requires Fe for essential metabolic pathways. Like several other pathogenic bacteria, it has evolved an intricate mechanism of acquiring, assimilating, and storing Fe, which is a component that determines the fate of the pathogen inside the host [28]. Because Fe is not freely available in the host, Mycobacteria must actively compete for this metal to establish an infection, but they must also carefully control Fe acquisition, as excess free Fe can be extremely toxic. The molecules responsible for Fe acquisition in mycobacteria include simple molecules such as salicylic acid and citric acid, apart from the two classes of siderophores.

To acquire Fe, mycobacteria produce siderophores (high-affinity Fe chelators). The lipophilic siderophores that remain associated with the cell wall are called mycobactins, and the second class of siderophores includes polar forms that are released into the extracellular medium [28]. These are called carboxymycobactins (released by pathogenic mycobacteria) and exochelins (released by nonpathogenic mycobacteria).* M. tuberculosis* and* M. smegmatis* produce salicylate-containing siderophores known as mycobactins. There are two forms of mycobactins: carboxymycobactin, which is a water-soluble secreted molecule, and the cell-associated mycobactin, which is a hydrophobic molecule that is retained on the cell surface. In addition to mycobactins,* M. smegmatis* also produces a peptidic siderophore known as exochelin, which is the predominant siderophore secreted by this mycobacterium under Fe limitation [28].

The identification of two genes that are annotated as fecB and fecB2 and that code proteins similar to FecB of* Escherichia coli* suggests that* M. tuberculosis* may also utilize ferric dicitrate as an Fe source [61]. Siderophores avidly bind Fe^+3^ and can effectively compete with host Fe binding proteins for this metal. Fe^+3^-carboxymycobactin can transfer Fe^+3^ to mycobactin or bring it into the cell via the iron-regulated transporter IrtAB. The putative transporter encoded by fxuABC may transport Fe^+3^-exochelin complexes.

Previous work has linked the ESX-3 system with the ability of mycobacteria to adapt to Fe limitation. ESX-3 is one of the five type VII secretion systems encoded by the* M. tuberculosis* genome. Studies that examined an* M. smegmatis* exochelin synthesis mutant indicated an ESX-3 requirement for Fe^+3^-mycobactin utilization. The precise role of ESX-3 in Fe acquisition in* M. tuberculosis* is unknown, but it is clear that ESX-3 is necessary for adaptation to low Fe conditions [62]. On the other hand, it has been documented that* M. tuberculosis* increases microvesicles production in response to Fe restriction and that these microvesicles contain mycobactin, which can serve as an iron donor and supports replication of Fe-starved mycobacteria. Consequently, the results revealed that microvesicles play a role in Fe acquisition in* M. tuberculosis*, and this can be critical for survival in the host. Recent studies have demonstrated that failure to assemble the Fe acquisition machinery or to repress Fe uptake has deleterious effects for* M. tuberculosis* [28].

A protein that was speculated to be a mycobacterial iron transporter is the Mramp, and this protein was able to increase the uptake of Fe^2+^ and Zn^2+^ in a pH dependent manner. Mramp was expected to be a cation transporter with no selective transport of Fe, although additional reports indicate that Mramp may act as a cation efflux pump [63].

Bacterioferritin-like molecules bfrA (a putative bacterioferritin) and bfrB (an Ft-like protein) have been identified in the* M. tuberculosis* genome and are the principal Fe storage molecules. Their expression is induced under Fe-rich conditions and repressed under Fe-deprived conditions. Therefore, it is speculated that this format allows the maintenance of basal levels of bacterioferritin inside the pathogen so that any amount of excess Fe can be immediately stored in a bound form [64]. Regulation of gene expression in* M. tuberculosis* includes that of regulatory proteins, stress response proteins, enzymes, and PE-PGR/PPE proteins. The genes that are upregulated under Fe-deprived conditions included those that are responsible for acquisition of Fe, such as siderophores, biosynthesis gene clusters mbt1 and mbt2, and Fe regulated transporters of siderophores irtA, irtB, Rv2895c, and esx [28]. Genes that are upregulated under Fe-rich conditions include bacterioferritin and ferritin (bfrA and bfrB), as they serve to store excess Fe as catalase-peroxidase, or katG and its regulator, ferric uptake regulator A (FurA) [63].

There are two Fur proteins, FurA and FurB. After binding ferric iron, FurA recognizes and binds to a 19-base-pair pseudopalindrome sequence of a specific DNA motif called Fur Box that is present upstream to a gene and acts as a repressor. FurB, on the other hand, was later found to be regulated by zinc and not Fe and has been correctly referred to as Zur.

IdeR, an Fe-dependent repressor and activator, is the major regulatory protein involved in homeostasis in mycobacteria. Belonging to the Diphtheria toxin repressor family (DtxR), it acts as a homodimer, with each monomer possessing two binding sites for Fe. Two homodimers with four bound Fe ions recognize a 19-base-pair palindromic sequence and in Fe-replete conditions and negatively regulate the expression of proteins required in Fe-depleted conditions [65]. The genes or gene clusters essentially required during Fe starvation are effectively repressed by IeR. These include the siderophore synthesis gene cluster, mbt1, mbt2, irtA, irtB, and Rv2895c. Therefore, there are certain proteins that are differentially regulated by Fe in an IdeR-independent fashion. These include lipoprotein IprE, KatG, 50S ribosomal protein, L22, and ATP synthase c chain, two component response regulators, MTrA, PE-PGRS proteins, and NifU-like proteins [28]. Fur and Fe-dependent repressors and activators or IdeR are the two key proteins that regulate expression of other Fe-dependent genes [28, 63].

### 4.10. *Candida* spp

*Candida* is a genus of yeast and is the most common cause of fungal infection worldwide [66, 67]. Many* Candida* species are harmless commensals or endosymbionts of hosts including humans; however, when mucosal barriers are disrupted or the immune system is compromised they can invade tissues and cause disease [66]. Among* Candida* species,* C. albicans* is responsible for the majority of Candida bloodstream and mucosal infections. However, in recent years, there is an increasing incidence of infections caused by* C. glabrata* and* C. rugosa*,* C. parapsilosis*,* C. tropicalis*, and* C. dubliniensis* [66]. Varied virulence factors and growing resistance to antifungal agents have contributed to their pathogenicity [66, 68].

*Candida albicans* can cause infections (candidiasis or thrush) in humans and other animals. Between the commensal and pathogenic lifestyles, this microorganism inhabits host niches that differ markedly in the levels of bioavailable iron. Once introduced into the bloodstream,* C. albicans* can acquire Fe from the molecules that are used by the host to sequester this metal [69]. For example, several groups have identified* C. albicans* hemolytic activity capable of releasing Hb from host erythrocytes. Free Hb or its heme/hemin metal-porphyrin ring is bound by a hemoglobin receptor, Rbt5, on the fungal cell surface, followed by endocytosis of Rbt5-hemoglobin complexes and release of Fe^2+^ by the heme oxidase, Hmx1 [69]. It has been reported that* C. albicans* encodes four additional homologs of Rbt5, of which Rbt51 has also been demonstrated to bind to hemin [69].

*C. albicans* can also utilize host Tf* in vitro* as a sole source of Fe, probably through the involvement of a transferrin receptor, similar to certain bacterial pathogens. It has been reported that the Fe^3+^ derived from Tf is taken up by a reductive iron uptake system that is conserved with the well-described high affinity iron uptake system of* Saccharomyces cerevisiae*. Fe^3+^ is first reduced to soluble Fe^2+^ by a cell surface-associated ferric reductase [69]. In coupled reactions, Fe^2+^ is then oxidized and imported into the fungal cytoplasm by a multicopper ferroxidase/iron permease complex.* C. albicans* encodes 17 putative ferric reductases, five putative multicopper ferroxidases, and four putative ferric permeases with potential functions in reductive Fe uptake, and different subsets of these enzymes are expressed under different* in vitro* conditions. Of the two ferric permeases, only Ftr1 is expressed when iron is limited, and FTR1 is essential in a murine bloodstream infection model of virulence [69].

**Figure 3:**
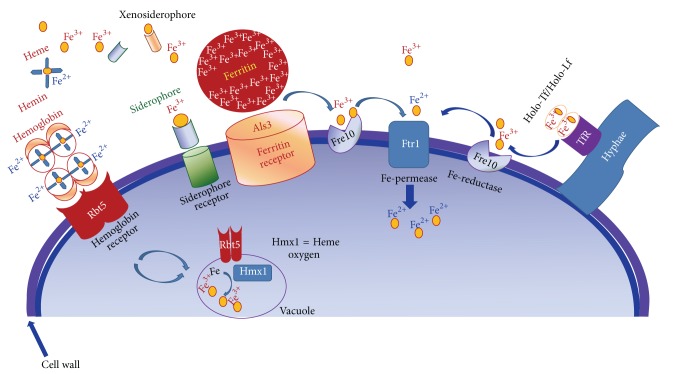
Iron acquisitions systems in* Candida albicans*. To acquire iron,* C. albicans* possesses three high-affinity iron acquisition systems: (1) a reductive system responsible for iron exploitation from transferrin or ferritin or from the environment; (2) a siderophore uptake system responsible for iron acquisition from a range of siderophores produced by other organisms; and (3) a heme-iron uptake and degradation system capable of acquiring iron from hemoglobin and probably from heme-proteins.

In tissues, the Fe is mainly bound to Ft. The Ft is found inside of macrophages and epithelial cells. This protein binds 4500 Fe atoms, and cytoplasmic iron-ferritin complexes are generally extremely stable. It has been documented that* C. albicans* utilizes Ft as Fe source* in vitro*, or directly from epithelial cells in culture. When this yeast was cocultured with a human oral epithelial cell line, the protein Ft was found bound onto their surface. This Ft binding protein denominated Als3, is located in the hyphae from* C. albicans* [69]. Als3 also plays important roles in* C. albicans* biofilm formation [70] and adhesion to host epithelial and endothelial cells and induced endocytosis of hyphae [71]. Thus, Als3 integrates Fe uptake and virulence functions but only in oral epithelial infection models. This conclusion was obtained when deletion of ALS3 abrogated* C. albicans* virulence in the oral epithelial infection model, but not in a bloodstream infection model [69, 72]. Additionally, it has been reported that,* in vitro*, fungal-mediated acidification of the laboratory culture media is required to dissociate Fe^3+^ from ferritin. Fe^3+^ is transported into the fungal cytoplasm via the same reductive Fe uptake system described above for Ft [69].  Figure 3 shows the iron acquisitions systems in* C. albicans*.

*C. albicans* also possesses a third system of iron uptake based in the use of siderophores; however, it is unclear whether* C. albicans* synthesizes its own siderophores. Siderophore activity has been reported for this species but its genome does not encode the known fungal biosynthetic enzymes [69, 73]. Nevertheless,* C. albicans* has been demonstrated to utilize exogenous ferrichrome-type siderophores via the Sit1 siderophore importer. Similar to ALS3, deletion of SIT1 abolishes* C. albicans* virulence in a reconstituted human epithelial infection model but not in a bloodstream infection model [69, 74]. Finally, it has been recently reported that Hap43, Sfu1, and Tup1 act coordinately and regulate iron acquisition, iron utilization, and other iron-responsive metabolic activities in* C. albicans* [75].

*Candida glabrata* is both a human fungal commensal and an opportunistic pathogen. It is the second most common cause of infection, surpassed only by* C. albicans*. This yeastis an intracellular pathogen that can survive phagocytosis and replicates within the host cell.* C. glabrata* infection is extremely difficult to treat due to its intrinsic antifungal resistance to azoles. The infections caused by this fungus are associated with a high mortality rate. Siderophore production is common among most microorganisms and is a major mechanism of Fe solubilization and acquisition. The very high Fe-binding contact observed for siderophores of fungal origin is approximately 10^30^ M at pH 7. Several bacteria and fungi do not produce siderophores but have evolved transporters that allow them to utilize siderophores they themselves do not produce. These are called xenosiderophores [76].

Computational analysis of Sit1 identified sequence signatures that are characteristic of members of the Major Facilitator Superfamily of Transporters. In a study by Nevitt and Thiele (2011), Sit1 is described as the sole siderophore Fe transporter in* C. glabrata*, and the study demonstrates that this siderophore is critical for enhancing their survival in the face of the microbicidal activities of macrophages [77]. Within the Sit1 transporter, a conserved extracellular siderophore transporter domain (SITD) was identified that is important for the siderophore-mediated ability of* C. glabrata* to resist macrophage killing and is dependent on macrophage Fe status [77]. They suggested that the host's iron status is a modifier of infectious disease that modulates the dependence on a distinct mechanism of microbial Fe acquisition. Iron-regulated CaSit 1 shares high homology with* S. cerevisiae* siderophore transporters and its deletion compromises utilization of fungal ferrichrome-type hydroxamate siderophores. The absence of an identifiable heme receptor in* C. glabrata* suggests that this pathogen may rely predominantly on the solubilization of the circulating exchangeable Fe pool to meet its requirements for Fe [76].

A study realized by Srivastava et al. (2014) described the molecular analysis of a set of 13* C. glabrata* strains that were deleted for proteins and potentially implicated in Fe metabolism. The results revealed that the high-affinity reductive Fe uptake system is required for the utilization of alternate carbon sources and for growth under both* in vitro* Fe-limiting and* in vivo* conditions. Further, they showed for the first time that the cysteine-rich CFEM domain-containing cell wall structural protein CgCcw14 and the putative hemolysin CgMam3 are essential for maintenance of intracellular Fe content, adherence to epithelial cells, and virulence [78]. Additionally, they present evidence that the mitochondrial frataxin CgYfh1 is pivotal to Fe metabolism and conclude that high-affinity iron uptake mechanisms are critical virulence determinants in* C. glabrata* [78].

### 4.11. *Cryptococcus neoformans*

*Cryptococcus neoformans* is a fungal pathogen and a leading cause of pulmonary and central nervous systemic mycosis in immunocompromised individuals such as HIV-infected patients. For this reason,* C. neoformans* is sometimes referred to as an opportunistic fungus. It is a facultative intracellular pathogen. In human infection,* C. neoformans* is spread by inhalation of aerosolized spores (basidiospores) and can disseminate to the central nervous system where it can cause meningoencephalitis [79]. In the lungs,* C. neoformans* are phagocytosed by alveolar macrophages. Macrophages produce oxidative and nitrosative agents, creating a hostile environment, to kill invading pathogens. However, some* C. neoformans* can survive intracellularly in macrophages. Intracellular survival appears to be the basis for latency, disseminated disease, and resistance to eradication by antifungal agents [80]. One mechanism by which* C. neoformans* survives the hostile intracellular environment of the macrophage involves upregulation of expression of genes involved in responses to oxidative stress.* C. neoformans* has been considered an excellent model fungal pathogen to study iron transport and homeostasis because of its intriguing connection with virulence. Growing evidence suggests that the fungus is able to utilize several different iron sources available in the host, and that the intracellular or extracellular localization of the pathogen influences its iron acquisition strategy [80].* C. neoformans* infects alveolar macrophages; at this site, specifically in the acidic phagolysosome, free Fe^2+^ is released from the host Ft and Tf. The reductive high-affinity Fe uptake system mediated by Cft1 and Cfo1 was characterized, its function was closely associated with the reduction of Fe^3+^ at the cell surface by the reductase activity, and it was limited in the environment at neutral pH [79].

**Figure 4:**
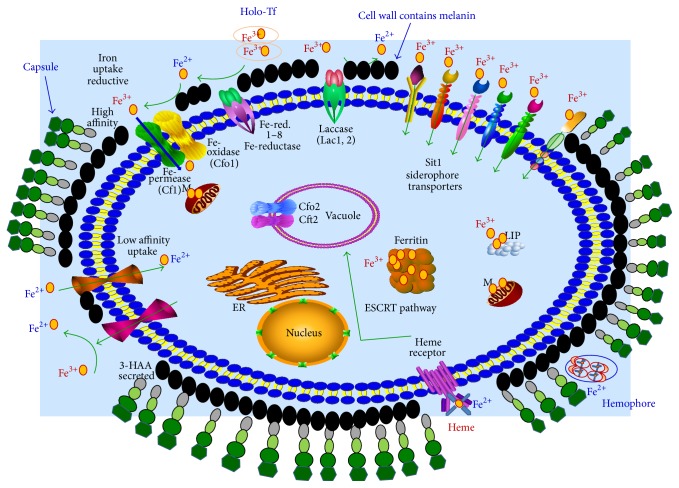
Iron acquisitions systems in* Cryptococcus neoformans*.* C. neoformans* infects alveolar macrophages; at this site, specifically in the acidic phagolysosome, free Fe^2+^ is released from the host Ft and Tf. The reductive high-affinity Fe uptake system mediated by Cft1 and Cfo1 plays a role in the reduction of Fe^3+^ at the cell surface by the reductase activity. In addition,* C. neoformans* is able to utilize Tf through the reductive high-affinity iron uptake system.Finally, for extracellular heme acquisition,* C. neoformans* relies on the complex Cft1/Cfo1, a xenosiderophore transporter (Sit1), and secreted and extracellular reductants (3-hydroxyanthranilic acid, melanin).

Therefore,* C. neoformans* could predominantly use an iron uptake system that is specifically responsive to the acidic intracellular niche, although Fe deprivation at an acidic pH no longer reduced the growth of the cft1 and cfo1 mutants. Moreover, a mutant lacking either CFT1 or CFO1 displayed attenuation of virulence and eventually caused disease in infected mice. These observations suggest that an as-yet unknown Fe uptake system, which is independent of the reductive high-affinity iron uptake system, may play a role in the acidic host microenvironment in a phagolysosome [79]. On the other hand,* C. neoformans* is able to utilize Tf through the reductive high-affinity iron uptake system and extracellular heme by Cig1 and the ESCRT complex; however, more studies should be carried out to understand how* C. neoformans* directly liberates Fe from Tf as well as Hb and other heme-containing proteins [80]. It has been suggested that the gene CIR1 (*Cryptococcus* iron regulator) shares structural and functional features with other fungal GATA-type transcription factors for iron regulation [81].  Figure 4 shows the iron acquisitions systems in* C. neoformans*.

### 4.12. *Leishmania* spp

Leishmaniasis is endemic in the tropics and neotropics. Clinical manifestations include skin lesions ranging from small cutaneous nodules to gross mucosal tissue destruction. The infection is transmitted to human beings and animals by sandflies.* Leishmania* parasites have a digenetic life cycle, alternating between the promastigote stage in the insect gut and the amastigote stage in macrophages of mammalian hosts. It has been postulated that* Leishmania* cells are equipped with diverse Fe acquisition mechanisms and are capable of utilizing various Fe sources, suggesting that Fe acquisition is essential for pathogenicity and that Fe deprivation could be an effective strategy for controlling leishmanial infections [82].

Like many other intracellular pathogens,* Leishmania* must be capable of acquiring Fe from the host milieu in order to thrive. In addition to Tf, the growth and survival of* L. infantum* and* L. amazonensis* amastigotes can be supported by Fe derived from hemoglobin and hemin [83]. The uptake of heme by intramacrophagic* L. amazonensis* amastigotes is mediated by the* Leishmania* heme response 1 (LHR1) protein. Furthermore, intracellular* L. amazonensis* also possesses a ferric reductase, the* Leismania* ferric iron reductase 1 (LFR1), which provides soluble Fe^2+^ for transport across the parasite plasma membrane by the ferrous iron transporter,* Leishmania* iron transporter 1 (LIT1) [83, 84]. Moreover, LIT1-mediated Fe acquisition seems to be essential for the differentiation of* L. amazonensis* parasites from the sandfly promastigote form to the macrophage-adapted amastigote form [85].

Apart from the mechanisms of direct iron internalization,* Leishmania* parasites can also subvert the host's Fe uptake systems to their own advantage. In fact,* L. amazonensis* amastigotes can obtain Tf by forcing the fusion of Tf-containing endosomes with the parasitophorous vacuole [86]. Alternatively,* L. donovani* is capable of decreasing the macrophage's labile Fe pool, a process that triggers an increased surface expression of transferrin receptor 1 and internalization of Tf, thus permitting continuous provision of Fe to the parasite. This decrease in the labile Fe pool of activated macrophages has recently been proposed to be the result of the downregulation of the expression of SLC11A1 by a* L. donovani*-secreted peroxidase. Also, in line with these data, it has been reported that the expression of ferroportin is downregulated in the spleen of* L. donovani*-infected mice, which may contribute to an increased accumulation of iron inside macrophages. In* Leishmania*, a transferrin receptor-based mechanism for Fe uptake was also initially postulated, but this mechanism was not confirmed by subsequent studies [87]. Tf can reach the lysosome-like parasitophorous vacuoles where Leishmania resides in macrophages, but it appears to function mainly as a source of Fe^3+^ for the sequential action of two surface-associated parasite molecules: the Fe^3+^ reductase LFR1 and the LIT1 transporter, which directly promote Fe^2+^ uptake. Intriguingly, the* T. cruzi* genome does not contain an obvious LIT1 orthologue, raising the possibility that this Fe^2+^-transporter represents a specific* Leishmania* adaptation to the low Fe environment of phagolysosomes [88]. Mutations in the lysosomal Fe efflux pump NRAMP1 confer susceptibility to* Leishmania* and other intravacuolar pathogens, reinforcing the conclusion that* Leishmania* needs a high-affinity transporter such as LIT1 to compete effectively for Fe within its parasitophorous vacuole [89]. On the other hand,* L. amazonensis* directly interferes with the Fe export function of macrophages, by inhibiting cell surface expression of Fpn1, but the mechanism by which this is achieved is still unknown [90].

### 4.13. *Trypanosoma* spp

The amastigotes of the intracellular parasite* Trypanosoma* cruzi take up Fe-loaded Tf when grown* in vitro*, but the physiological significance of this process is unclear [91]. Tf is restricted to the lumen of the endocytic pathway and is therefore absent from the host cell cytosol, where intracellular amastigotes replicate. The bloodstream form of* Trypanosoma brucei* acquires Fe from Tf by receptor-mediated endocytosis by a process that is regulated by Fe availability. TrR is a heterodimeric complex encoded by two expression site-associated genes, ESAG6 and ESAG7, and shares no homology with the homodimeric mammalian Tf receptor. The binding of one molecule of Tf requires the association of both ESAG6 and ESAG7. In mammalian cells, the TfR mRNA is stabilized in iron-depleted cells due to the binding of IRPs to specific IREs. In* T. brucei*, this IRP-1 relation is not essential for Fe regulation of ESAG6 mRNA. In mammalian cells, the closely related IPR-2 can independently mediate the iron status via IREs. However, in trypanosomes, the presence of additional IRP-related proteins seems very unlikely. The* T. brucei* genome contains only one IRP-related gene, which suggests that a different mechanism, a different type of transacting factor, is responsible for Fe sensing and regulation of transferrin receptor mRNA in this protozoan [91, 92]. However, it is unknown how procyclic forms that cannot bind Tf acquire Fe. Additionally, the bloodstream-form of* T. brucei* acquires Fe by receptor-mediated endocytosis of host transferrin [93]. The mechanism(s) by which Fe is then transferred from the lysosome to the cytosol remains unresolved [94].

## 5. Conclusions

The use of Fe as a cofactor in basic metabolic pathways is essential to both pathogenic microorganisms and their hosts. It is also a pivotal component of the innate immune response through its role in the generation of toxic oxygen and nitrogen intermediates. During evolution, the shared requirement of micro- and macroorganisms for this important nutrient has shaped the pathogen-host relationship [14]. Two general mechanisms of Fe acquisition in intracellular parasites have been described: siderophore-mediated Fe acquisition by cognate receptors and receptor-mediated Fe acquisition from host Fe-binding proteins [14]. Intracellular microorganisms have evolved a variety of siderochromes, which are special ligands that can dissolve insoluble Fe^3+^ and facilitate its transport into the cell in order to acquire Fe from Tf and other Fe-proteins in the host. The success of intracellular parasites seems to be related mainly to their ability to take up Fe from the protein Tf [12]. Once ingested by macrophages, intracellular parasites are taken up by phagosomes via endocytosis. Acidification of the phagosome permits the iron to be released from Tf, and, in this way, some pathogens can gain access to this element [12].

Bacteria use the protein ferritin or bacterioferritin to store Fe. These are ubiquitous Fe storage proteins that play a fundamental role in cellular Fe homeostasis and have similarities with Ft that is found in mammals. Bacterial Fts have the capacity to store very large amounts of Fe as a Fe^3+^ mineral inside its central cavity. In times of Fe deprivation, some bacteria require that iron be released from Ft mineral stores in order to maintain their metabolic rate and growth. In times of Fe repletion, intracellular microorganisms must regulate the genes required for Fe acquisition, but this mechanism has not been fully characterized [45, 61]. Transferrin and its receptor (TfR1) play an important role during infection of macrophages with bacterial pathogens that prefer an intracellular lifestyle. Expression of TfR1 can in turn be modulated by bacterial infections. Some pathogens actively recruit TfR1 to the bacterium-containing vacuole [29, 45].

The notion is conceivable that intracellular pathogens reside in phagosomal compartments to modulate Fe regulatory proteins, thereby increasing their Fe availability, but this notion is still speculative. The Fe acquisition process often begins when cell surface receptors recognize Fe^3+^ complexes and ultimately ends when cytoplasmic membrane (CM) transporters internalize and, in some cases, reduce the metal to Fe^2+^, which then enters cytoplasmic metabolic pools [14]. Despite many advances, the exact role of Fe acquisition systems* in vivo* and their effects in pathogenic virulence remain to be determined.

## Figures and Tables

**Table 1:** Iron sources and mechanisms of uptake, transport, and regulation used by intracellular parasites.

Parasite	Iron sources	Mechanisms of iron acquisition	Transport	Regulation
*Francisella tularensis *	Tf	Receptors (TfR1)	Ferrireductase/DMT-1	IRP1-IRP2
	Fe^3+^	iron reductase, siderophores	Fs1E	
	Fe^2+^	Receptors, iron reductase?	FupA	
	Lf	Receptors, iron reductase?	Ferrireductase/DMT-1?	

*Salmonella* spp.	Heme	Heme-oxygenase-1?	Porins?	FtnA, FtnB, FtnC, FtnD
	Tf	siderophores (enterobactin,	FepBCDG	sitABCD/Fur
	Fe^3+^	enterochelin)/IronN and FepA	Feo	
	Fe^2+^	Phagocytosis		

*Chlamydia *	Tf	Receptors?	tonB analogue?	IRP-1/IRE?
	Ft	Receptors?, iron reductase?	Host Fe-transport pathways?	
		Receptors?	ABC transport systems?	
		Siderophores?		

*Neisseria *spp.	Tf	Receptors (TbpA, TbpB)	FetA	Fur
	Lf	Receptors (LbpA, LbpB)	MpeR	
	haptoglobin-Hb	Siderophores (enterobactin Salmochelin)	HmbR	

*Legionella pneumophila *	Fe^3+^	Siderophores	Feo AB/system	Fur?
	Fe^2+^	iron reductase	PerR/Fur	
	Tf and Ft?	iron reductase?	Lpp_2867	

*Shigella *spp.	Tf	Siderophores	Feo	ArcA and FNR
	Fe^3+^, Fe^3+^	iron reductase	Feo, Sit, luc	Fur
	Heme	iron reductase	Shu	
	LF			

*Listeria monocytogenes *	Hb	HupDGC	Fhu	Fri/Fur
	Hemin	HupDGC	Fhu	PerR
	Ferric citrate	iron reductase	Ferric citrate systems	
	Fe-proteins?	Siderophores, iron reductase	Fur, ABC transporters	

*Coxiella burnetii *	?	?	?	Bacterioferritins
				Fur

*Mycobacterium *spp.	Ferric dicitrate	Salicylic acid	IrtAB	Bacterioferritins
	Tf, Lf, Ft	Citric acid	Mramp	FurA, FurB
		Siderophores		IdeR

*Candida *spp.	Heme	Heme-binding protein (Dap1)	Sit1 (iron transporter)	
	Tf Ft	Fe^3+^ reductases, Fe^2+^transporter		Sfu1
	Hemin			

*Cryptococcus neoformans *	Tf	Iron reductase, iron permeases	Cft1 and Cfo1	Cir1
	Heme	Hemophores, Heme receptors	Cig1 and the ESCRT	
	Ft			

*Leishmania *spp.	Heme, Hemin	LHR1	LIT1 transporter	?
	Tf?	Fe^3+^ reductase 1 (LFR1),		
	Lf			

*Trypanosoma *spp.	Tf	Receptors (TfR)/endocytosis	ESAG6, ESAG7	IRP/IRE
